# Comment on: “Do maternal albumin levels affect post-operative complications after cesarean delivery?”

**DOI:** 10.1186/s12884-023-05756-6

**Published:** 2023-06-14

**Authors:** Shuangqiong Zhou, Xiuhong Cao, Zhiqiang Liu

**Affiliations:** Department of Anesthesiology, Shanghai Key Laboratory of Maternal Fetal Medicine, Shanghai Institute of Maternal-Fetal Medicine and Gynecologic Oncology, Shanghai First Maternity and Infant Hospital, School of Medicine, Tongji University, Shanghai, 200092 China

**Keywords:** Serum albumin, Cesarean delivery, Postoperative outcome, Surgical site infection

## Abstract

Hypoalbuminemia is often considered an independent risk factor for surgical site infections. This study first demonstrated that albumin level ≥ 3.3 g/dL was independently associated with adverse maternal outcomes. In this letter to the editor, we would like to raise some concerns about the study and clarify the interpretation of the results.

## Main text

Surgical site infection (SSI) is a major cause of prolonged hospital stay and a burden to the healthcare system [1, 2]. Maternal SSI after cesarean delivery is a clinical problem that contributes to significant morbidity and mortality rates. We read with great interest the study by Yagur et al., who published a retrospective cohort study, the first to analyze the relationship between maternal serum albumin levels before elective cesarean delivery (CD) and postoperative complications [3]. The results suggested that high serum albumin levels among women undergoing CD might be associated with abnormal postoperative outcomes. However, the results of the study were unexpected; based on these results, it can be hypothesized that changes in serum albumin level, especially increases from the normal range, may affect maternal pregnancy outcomes.

In this retrospective cohort study, many risk factors for SSI were included, However, many other relevant variables likely were not measured. Since both groups differed significantly across measured variables at admission, it is likely that unmeasured variables were similarly unbalanced. We think it is too early to come to the conclusion and offer the following points as counter-argument.

First, in this article, parturients with preeclampsia were excluded from the study. Tran et al. [4] found that preeclampsia was an independent risk factor for SSI. Preeclampsia is often associated with hypoalbuminemia. Thus, the exclusion of women with preeclampsia may influence the results.

Second, risk factors such as surgery duration, [5] hypertension disorders, [6] and blood transfusion during CD were not included. Prolonged operative time has been shown to increase the risk of SSI [7]. Furthermore, hypertensive disorder is an independent risk factor for wound infection after CD [6]. In a hospital-based case-control study, blood transfusion during or following delivery, was shown to be an independent risk factor for SSI [8]. Mazzeffi et al. declared that RBC transfusion may be associated with increased risk for organ space SSI and septic shock after colon resection surgery [9].

Third, obesity, especially pregestational obesity, as a potential risk for post-CD wound infection has been discussed previously [4–6, 10–12]. Maternal obesity (body mass index (BMI) ≥ 30 kg/m^2^) was significantly linked to an increased incidence of SSI. While pregestational BMI was not included in the article, delivery BMI was divided into BMI < 25 kg/m^2^ and BMI ≥ 25 kg/m^2^. Thus, the proportion of BMI > 30 kg/m^2^ was not compared between the two groups. Furthermore, other factors such as incision length > 16.6 cm [10] and subcutaneous tissue thickness > 3 cm, [13] that have a great effect on the risk of developing SSI were not considered. Diabetes mellitus was included in this study, but it was unclear whether this was gestational diabetes mellitus, which could have affected the results.

Finally, we have a few concerns regarding the methodology of this study. First, a receiver operating characteristic (ROC) curve was generated, and a cutoff value of 3.3 g/dL was provided in this study. However, the ROC curve was not presented and neither the value of sensitivity, specificity, nor the area under the ROC curve and its 95% confidence interval were described. Secondly, the presented regression is composed of many variables, but there is no explanation why these variables were entered as no description of the groups is presented. As this study was to analyze whether high serum albumin levels among women undergoing CD affect risks of postoperative outcomes, a case-control study may be more appropriate which begins with an outcome to comprehend the cause.

In conclusion, many details about the course of deliveries are unavailable in this study. The result may only be convincing after accounting for these confounding factors. There are multiple possible causes of SSI that the study does not address.

## Data Availability

Not applicable.

## Abbreviations

SSI: Surgical site infection
CD: cesarean section
BMI: body mass index
